# Is Fetal Growth Restriction Associated with a More Severe Maternal Phenotype in the Setting of Early Onset Pre-Eclampsia? A Retrospective Study

**DOI:** 10.1371/journal.pone.0026937

**Published:** 2011-10-28

**Authors:** Jane Weiler, Stephen Tong, Kirsten R. Palmer

**Affiliations:** 1 Department of Obstetrics and Gynaecology, Monash Medical Centre, Clayton, Victoria, Australia; 2 Translational Obstetric Group, Department of Obstetrics and Gynaecology, University of Melbourne, Mercy Hospital for Women, Heidelberg, Victoria, Australia; University of Southampton, United Kingdom

## Abstract

**Background:**

Both pre-eclampsia and fetal growth restriction are thought to result from abnormal placental implantation in early pregnancy. Consistent with this shared pathophysiology, it is not uncommon to see growth restriction further confound the course of pre-eclampsia and vice versa. It has been previously suggested that superimposed growth restriction is associated with a more severe pre-eclamptic phenotype, however this has not been a consistent finding. Therefore, we set out to determine whether the presence of fetal growth restriction among women with severe early-onset pre-eclampsia was associated with more severe maternal disease compared to those without a growth-restricted fetus.

**Methods and Findings:**

We undertook a retrospective cohort study of women presenting to a tertiary hospital with severe early-onset pre-eclampsia (<34 weeks' gestation) between 2005–2009. We collected clinical data, including severity of pre-eclampsia, maternal and neonatal outcomes. Of 176 cases of severe pre-eclampsia, 39% (n = 68) were further complicated by fetal growth restriction. However, no significant difference was seen in relation to the severity of pre-eclampsia between those with or without a growth-restricted baby. The presence of concomitant growth restriction was however associated with a significantly increased risk of stillbirth (p = 0.003) and total perinatal mortality (p = 0.02).

**Conclusions:**

The presence of fetal growth restriction among women with severe early-onset pre-eclampsia is not associated with increased severity of maternal disease. However the incidence of stillbirth and perinatal death is significantly increased in this sub-population.

## Introduction

Pre-eclampsia is a condition that affects 5–8% of all pregnancies and is responsible for a significant proportion of global maternal and perinatal morbidity and mortality [1]. The pathophysiology has been shown to relate to abnormal shallow trophoblast invasion of the maternal uterine spiral arteries in early pregnancy [2]. This results in a reduction in the amount of blood flow to the utero-placental bed leading to a persistent state of placental hypoxia through-out pregnancy [3]. In response to this hypoxic state the placenta releases a number of anti-angiogenic factors into the maternal circulation that result in widespread endothelial dysfunction [4]. It is this endothelial dysfunction that produces the clinical characteristics of pre-eclampsia, being hypertension, renal dysfunction and proteinuria, liver impairment, thrombocytopenia, and increased vascular permeability with oedema affecting the periphery, brain and liver.

The complexity of this system becomes apparent as similar placental changes are also seen in pregnancies complicated by fetal growth restriction (FGR) without co-existent pre-eclampsia [5]. Furthermore, an elevation in anti-angiogenic factors soluble fms-like tyrosine kinase 1 (sFlt-1) and soluble Endoglin (sEng) are also seen in such pregnancies, albeit to a lesser extent [6], [7], [8].

These findings are in keeping with the long held belief that both IUGR and pre-eclampsia are likely part of a similar pathophysiological spectrum, being abnormal placental implantation in early pregnancy [9]. This premise is further strengthened by the significant overlap in their clinical presentations, with evidence suggesting that 12.8–58.6% of women with severe early onset pre-eclampsia will be further complicated by the presence of fetal growth restriction [10], [11]. Conversely, it has been shown that approximately 15% of women diagnosed with FGR will be diagnosed with superimposed pre-eclampsia later in the pregnancy [12].

Of clinical interest, several studies have proposed that the combination of pre-eclampsia with FGR is associated with a more severe pre-eclamptic phenotype when compared to women with pre-eclampsia and no FGR [12], [13]. Mitani et al. [12] examined 133 women between 22 weeks' gestation and term with pre-eclampsia with or without FGR. They found that women with pre-eclampsia and FGR had more severe hypertension and proteinuria, as well as higher rates of complications, such as placental abruption and disseminated intravascular coagulation. However this was not seen in a study by Haddad et al. [14] who found no significant difference in the severity of hypertension or rates of maternal complications between 239 women with early-onset pre-eclampsia either with or without a growth restricted fetus.

Given the heterogeneity of pre-eclampsia and the likelihood that the pathophysiology differs between early-onset and late-onset disease [15], it is important to study these populations separately. Here we undertake the first study among women with exclusively severe early-onset pre-eclampsia examining whether severity of the maternal disease is worse if there is co-existing FGR.

## Methods

### Objectives

The primary outcome was to determine whether the severity of pre-eclampsia differed depending on the presence or absence of FGR.

Secondary outcome measures related to whether there were any significant differences in the baseline characteristics, antenatal ultrasound assessments or neonatal outcomes between women with severe pre-eclampsia with or without FGR.

### Participants

All women who delivered between 2005 and 2009 at a tertiary Melbourne women's hospital with a pregnancy complicated by pre-eclampsia were identified from the hospital birthing outcomes summary database. The inclusion criteria were severe, early-onset pre-eclampsia requiring delivery <34 weeks'. Severe pre-eclampsia was defined according to the ACOG guidelines as the presence of one or more of: blood pressure ≥160/110 on two or more occasions, proteinuria ≥5 g/day, visual disturbance, oliguria, epigastric pain, liver dysfunction, thrombocytopenia or fetal growth restriction [16]. Gestational age was based on a first trimester ultrasound determination of estimated date of delivery (EDD) and/or accurate last menstrual period dates from regular menstrual cycles. Where a difference of greater than one week existed between the EDD derived from last menstrual period and first trimester ultrasound, then the date from the latter was chosen [17].

### Study Design

A retrospective cohort study was undertaken of 176 women with severe early-onset pre-eclampsia. Fetal growth restriction was defined as <10^th^ centile according to the Australian population infant birthweight chart [18]. When this was applied to our population of 176 women, 68 were identified as having severe pre-eclampsia and FGR. Outcomes were then compared between these women and the remaining 108 with severe pre-eclampsia and without FGR.

Baseline characteristics were obtained relating to maternal age, parity, body mass index (BMI), ethnicity, smoking status, alcohol consumption, gestation at delivery, birthweight and indication for delivery. The clinical features of pre-eclampsia were also recorded relating to maximal systolic and diastolic blood pressures pre-delivery, level of proteinuria, alanine transaminase (ALT), platelets, creatinine and uric acid, use of magnesium sulfate, use of anti-hypertensives and proportion remaining on anti-hypertensives at discharge.

Antenatal ultrasound findings for fetal wellbeing were compiled, including umbilical artery doppler results, amniotic fluid index (AFI) and estimated fetal weight (EFW).

Neonatal outcome data recorded was steroid administration pre-delivery, apgar scores at one and five minutes, cord lactates, stillbirth, total perinatal mortality, and rates of necrotising enterocolitis, intraventricular haemorrhage, respiratory distress syndrome and jaundice requiring phototherapy.

### Ethics

The Human Research Ethics Committee at Southern Health approved this study (application number 10157Q). In accordance with this approval for a retrospective analysis of patient data, no individual patient consent was required.

### Statistical Methods

Normally distributed continuous variables were analysed using an unpaired student's t test, with data expressed as mean (± standard error of the mean (SEM)). Categorical variables were assessed using a Fishers exact test and data expressed as number (percentage of population involved). Statistical analysis was performed using GraphPad Prism software (La Jolla, CA).

## Results

A total of 583 women diagnosed with pre-eclampsia were delivered between 2005–2009 at Monash Medical Centre. Of these, 179 were found to have delivered between 24 and 34 weeks' gestation. Clinical details were analysed, leading to the further exclusion of 3 women who did not meet the criteria for severe pre-eclampsia. From the remaining 176 women identified as having severe early-onset pre-eclampsia, 39% (n = 68) were also complicated by the presence of FGR. Of those with FGR 41% (n = 30/68) were less than the 5^th^ centile.

A comparison of women with severe early-onset pre-eclampsia both with or without FGR showed no significant differences in maternal age, primiparity, ethinicity, BMI, smoking status or use of alcohol during pregnancy, as outlined in Table 1. Similarly the majority of women in both cohorts delivered via caesarean section (90% without FGR vs. 86% with FGR) with the indication for delivery in both cohorts mainly being on fetal grounds (53% without FGR vs. 64% with FGR). Women with pre-eclampsia and FGR delivered infants with a significantly lower birthweight than those without FGR (968.2 g (±44.13) vs. 1481 g (±39.61); p<0.0001). Furthermore, those with FGR delivered more prematurely than those without FGR (30 weeks vs. 31 weeks; p = 0.02).

**Table 1:** Baseline Characteristics of women with pre-eclampsia with or without fetal growth restriction.

	PET Without FGR N = 108	PET With FGR N = 68	p-value
Maternal Age - Years	29.76 (±0.61)	30.21 (±0.68)	0.63
Primiparous	60 (56%)	44 (65%)	0.27
BMI (kg/m^2^)	27.97 (±0.58)	29.38 (±0.76)	0.14
Smoker	14 (14%)	9 (15%)	0.82
Alcohol Use	9 (10%)	4 (9%)	0.77
Gestation at Delivery - Weeks	31	30	0.02
Caesarean Delivery	100 (90%)	61 (86%)	0.48
Birthweight	1481 (±39.61)	968.2 (±44.13)	<0.0001
**Indication for Delivery**			
Fetal	58 (53%)	44 (64%)	0.16
**Ethinicity**			
White	83 (76%)	55 (81%)	0.53
African	3 (3%)	1 (1%)	0.58
Asian	14 (13%)	6 (9%)	0.25
Hispanic	2 (2%)	1 (1%)	0.86
Other	6 (5%)	5 (7%)	0.64

Data is shown as number (%) and mean (± standard error of the mean) where appropriate. Where FGR = fetal growth restriction, BMI = body mass index.

There were no significant differences in the severity of pre-eclampsia experienced between the two groups. Similar systolic/diastolic blood pressures were seen among those with FGR when compared to those without FGR (174.6/103.1 mmHg Vs. 177.3/105.6 mmHg; p = 0.22/0.18). The level of proteinuria also showed no significant differences between those with or without FGR (4.597 g/day Vs. 4.925 g/day; p = 0.70). A number of biochemical parameters were elevated among women complicated by pre-eclampsia and co-existent FGR compared to pre-eclampsia and no FGR, but none were statistically significant. This was evidenced by the level of platelets (192×10^9^/L Vs. 194.1×10^9^/L; p = 0.88), ALT (84.63 U/L Vs. 47.93 U/L; p = 0.09) and creatinine (83.44 µmol/L Vs. 79.66 µmol/L; p = 0.54) in those with versus without FGR, as shown in Table 2.

**Table 2:** Pre-eclamptic characteristics.

	PET Without FGR N = 108	PET With FGR N = 68	p-value
Maximum SBP	177.3 (±1.75)	174.6 (±3.02)	0.22
Maximum DBP	105.6 (±1.08)	103.1 (±2.54)	0.18
Use of MgSO_4_	43 (40.2%)	19 (29%)	0.14
Use of anti-hypertensives	104 (98%)	63 (95.4%)	0.37
Discharged on anti-hypertensives	87 (89%)	43 (77%)	0.06
Proteinuria (g/day)	4.925 (±0.54)	4.597 (±0.62)	0.70
ALT (U/L)	47.93 (±8.05)	84.63 (±24.05)	0.09
Platelets (×10^9^/L)	194.1 (±9.02)	192.0 (±10.80)	0.88
Creatinine (µmol/L)	79.66 (±3.58)	83.44 (±5.34)	0.54
Uric Acid (µmol/L)	423.6 (±8.75)	409.2 (±11.37)	0.31

Data is shown as number (%) and mean (± standard error of the mean) where appropriate. Where SBP = systolic blood pressure, DBP = diastolic blood pressure, ALT = alanine transferase.

Significant differences were found however on antenatal ultrasound assessments for fetal growth and wellbeing (Table 3). The placental function and degree of fetal compromise was assessed through the use of umbilical artery Doppler and amniotic fluid index measurements. Doppler analysis was abnormal with either raised, absent or reversed end-diastolic flow in 31% of women without FGR vs. 87% of women with FGR (p<0.0001). Similarly, 27% of women without FGR showed evidence of oligohydramnios vs. 57% of women with FGR (p = 0.003). Ultrasound has been shown to have good positive and negative predictive values in identifying FGR in women with severe pre-eclampsia [19]. Certainly within our study population, ultrasound biometry assessments proved to be relatively reliable in identifying women with FGR antenatally. 79% of women with pre-eclampsia who delivered a growth-restricted baby were diagnosed with intra-uterine growth restriction (IUGR) antenatally, compared to 11% without growth-restriction appearing IUGR antenatally (p<0.0001).

**Table 3:** Ultrasound Findings.

	PET Without FGR N = 108	PET With FGR N = 68	p-value
**Umbilical Artery Doppler**			
Raised	16 (19%)	18 (32%)	<0.0001
AEDF	9 (11%)	23 (41%)	<0.0001
REDF	1 (1%)	8 (14%)	<0.0001
**AFI**			
Oligohydramnios	22 (27%)	20 (57%)	0.003
**EFW**			<0.0001
<3^rd^ centile	1 (2%)	21 (40%)	
3^rd^–10^th^ centile	5 (9%)	20 (39%)	
>10^th^ centile	51 (89%)	11 (21%)	

Data is shown as number (%) and mean (± standard error of the mean) where appropriate. Where AEDF = absent end diastolic flow, REDF = reversed end diastolic flow, AFI = amniotic fluid index, EFW = estimated fetal weight, compared using a contingency table.

Assessment of neonatal outcomes showed significant differences in relation to stillbirth and total perinatal mortality rates. One stillborn baby occurred in the cohort without FGR vs. eight stillbirths among women with FGR (0.9% vs. 11.3%; p = 0.003). Similarly total perinatal mortality was lower in our non-FGR population compared to the FGR group (5.5% vs. 22.1%; p = 0.02). By comparison, no significant differences in the percentage of infants receiving steroids pre-delivery (96% without FGR vs. 90% with FGR) was seen, with babies born in both groups showing similar apgars and cord lactates at delivery. There was also no significant difference in perinatal morbidity with similar rates of necrotising enterocolitis, intraventricular haemorrhage, respiratory distress syndrome and jaundice requiring phototherapy seen between both groups (see Table 4).

**Table 4:** Neonatal Outcomes.

	PET Without FGR N = 108	PET With FGR N = 68	p-value
Apgar 1 min	6.6 (±0.19)	6.4 (±0.28)	0.55
Apgar 5 mins	8.3 (±0.14)	8.1 (±0.18)	0.21
IUGR<5^th^ Centile	0	30 (42%)	
IUGR 5^th^–10^th^ Centile	0	41 (58%)	
Steroids pre-delivery	104 (96%)	63 (90%)	0.11
Cord Lactate	3.6 (±0.21)	4.3 (±0.32)	0.06
Stillbirth	1 (0.9%)	8 (11.3%)	0.003
Perinatal Death	6 (5.5%)	15 (22.1%)	0.02
NEC	2 (1.8%)	3 (4%)	0.38
IVH	4 (3.5%)	5(6.6%)	0.49
RDS	69 (61%)	42 (58%)	0.65
Jaundice req phototherapy	98 (88%)	60 (82%)	0.39

Data is shown as number (%) and mean (± standard error of the mean) where appropriate. Where IUGR = intrauterine growth restriction, NEC = nectrotising enterocolitis, IVH = intraventricular haemorrhage, RDS = respiratory distress syndrome.

## Discussion

Our findings demonstrate that 39% of women with severe early-onset pre-eclampsia are further complicated by fetal growth restriction. The severity of maternal disease was not altered by the co-existence of FGR. This was not the case however for the neonate, as a significant increase in both the rates of stillbirth and total perinatal mortality was seen among the cohort of growth restricted babies compared to their appropriately grown counterparts.

It has been suggested that pre-eclampsia further complicated by FGR results in a more severe pre-eclamptic phenotype. Indeed, Mitani et al. [12] showed that women with pre-eclampsia and FGR were more likely to demonstrate significantly elevated hypertension and proteinuria compared to their pre-eclamptic counterparts carrying appropriately grown infants. In keeping with this the cohort affected by FGR had significantly elevated risks of abruption, coagulopathy and HELLP syndrome. However, a possible explanation for these findings could relate to the difference in gestational ages between those complicated by FGR compared to those without FGR in that study. The FGR group delivered on average at 32.6 weeks consistent with a diagnosis of early-onset pre-eclampsia. However, the non-FGR group delivered on average at 36.1 weeks. While this is still pre-term it is more consistent with late onset pre-eclampsia, which is often a less severe disease and is associated with less maternal risk [15]. More recently a number of studies have suggested that the presence of FGR does not alter the severity of maternal disease relating to pre-eclampsia [11], [14], [20], a finding that our research further supports.

The main advantage of our data in comparison to other contemporaneous studies [11], [14], [20] is that we focused solely on the severe early-onset pre-eclamptic population, the population group at highest risk of severe maternal morbidity and mortality. Secondly, we are the first to examine the severity of the pre-eclamptic disease as our primary outcome. Furthermore, we were potentially more thorough in reporting the pre-eclamptic phenotype by including a number of biochemical markers, anti-hypertensive use, maximal recorded blood pressure readings, magnesium sulfate requirements and detailed neonatal outcomes.

While an observational study, our findings that the severity of the maternal disease is not affected by the concomitant presence of FGR should provide some reassurance to treating clinicians. For women presenting with severe preterm pre-eclampsia, increasing evidence is promoting expectant management to prolong gestation to reduce the potential morbidity and mortality of prematurity for the infant [11], [14]. These findings suggest that the presence of FGR should not necessarily dissuade clinicians from expectant management on the grounds that they may be concerned that women will develop a more severe pre-eclamptic phenotype. However, given the significantly higher mortality seen for the neonate the suitability for expectant management should involve regular assessment of fetal wellbeing.

It was interesting to note that the majority of pregnancies with severe early onset pre-eclampsia complicated by FGR had ultrasonographic evidence of placental insufficiency. 87% of these pregnancies had abnormal umbilical dopplers and 57% had oligohydramnios. The precise mechanisms that cause abnormal umbilical dopplers and oligohydramnios are unknown. However, these pregnancies often display villous maladaption with reduced volumes of both villi and the intervillous space seen, changes that have been linked to subsequent fetal hypoxia [21], [22].

This study is limited by its retrospective design and relatively small sample size. However, given severe early onset pre-eclampsia complicates approximately 0.8% of all pregnancies [23] our cohort is still relatively large. Monash Medical Centre is a major referral centre meaning that we were able to capture a sizeable cohort. Furthermore, being an observational study the ability to translate the findings to a wider clinical audience is potentially limited. Given the significant impact seen for the neonate it would be of benefit to undertake a larger study to better understand the implications of this presentation for both the mothers and their babies.

This study highlights that while severe early-onset pre-eclampsia is an uncommon condition of pregnancy, concomitant FGR complicates a significant proportion. Of clinical reassurance, the presence of FGR was not associated with a more severe maternal pre-eclamptic presentation within our population, however higher neonatal mortality was seen. The exact underlying aetiology that results in their shared presentation remains the focus of ongoing research.
